# Aberrant expression of miR-21, miR-376c and miR-145 and their target host genes in Merkel cell polyomavirus-positive non-small cell lung cancer

**DOI:** 10.18632/oncotarget.11222

**Published:** 2016-08-11

**Authors:** Ismini Lasithiotaki, Eliza Tsitoura, Anastasios Koutsopoulos, Eleni Lagoudaki, Chara Koutoulaki, George Pitsidianakis, Demetrios A. Spandidos, Nikolaos M. Siafakas, George Sourvinos, Katerina M. Antoniou

**Affiliations:** ^1^ Department of Thoracic Medicine, University Hospital, Medical School, University of Crete, Heraklion 71110, Greece; ^2^ Laboratory of Cellular and Molecular Pneumonology, Medical School, University of Crete, Heraklion Crete 71110, Greece; ^3^ Laboratory of Clinical Virology, Medical School, University of Crete, Heraklion Crete 71110, Greece; ^4^ Department of Pathology, Medical School, University of Crete, Heraklion Crete 71110, Greece

**Keywords:** merkel cell polyomavirus, non-small cell lung cancer, microRNAs, signalling pathways

## Abstract

Merkel Cell Polyoma Virus (MCPyV) infection has been associated with non-small cell lung cancer (NSCLC). Viruses can manipulate cellular miRNAs or have a profound impact on cellular miRNA expression to control host regulatory pathways. In this study, we evaluated the expression profiles of cancer-associated and virally affected host microRNAs miR-21, miR-145, miR-146a, miR-155, miR-302c, miR-367 and miR-376c in a series of NSCLC tissue samples as well as in samples from “healthy” sites, distant from the tumour region that were either positive or negative for MCPyV DNA. miR-21 and miR-376c were significantly upregulated whereas miR-145 was significantly downregulated in the MCPyV+ve samples compared to the MCPyV-ve tumour samples. Overall, miR-21 and miR-376c expression was higher in tumour compared to healthy tissue samples. No association was observed between the miR-155, miR-146a, miR-302c and miR-367 levels and the presence of MCPyV. The expression of miR-21 target genes (*Pten, Bcl-2, Daxx, Pkr, Timp3)*, miR-376c (*Grb2, Alk7*, *Mmp9*) and miR-145 (*Oct-4, Sox2*, *Fascin1*) and their associated pathways (*Braf, Akt-1, Akt-2, Bax, Hif1a, p53*) was altered between MCPyV+ve tumor samples and their corresponding controls. These results show a novel association between miR-21, miR-376c and miR-145 and their host target genes with the presence of MCPyV, suggesting a mechanism of virus-specific microRNA signature in NSCLC.

## INTRODUCTION

Lung cancer remains the leading cause in cancer-related mortality in both males and females. Approximately 85% of lung tumors are non-small cell lung cancer (NSCLC), including adenocarcinoma, squamous cell carcinoma and large cell carcinoma [1]. Although the majority of lung cancer patients are smokers, only a minority among smokers will develop this disease, strongly suggesting that additional environmental determinants including virus infections, in a background of genetic susceptibility, drive disease initiation and progression [2].

The accumulation of genetic and epigenetic events in the respiratory epithelium is essential for lung carcinogenesis [3]. In particular, microRNAs (miRNAs) have been shown to be commonly dysregulated in lung cancer [4]. MicroRNAs, short, non-coding RNA molecules, are key regulators of transcription regulation and it is currently appreciated that they participate in tumorigenesis by regulating the expression of oncogenes and tumor suppressors or by acting as oncogenes and tumor suppressors themselves [5]. Furthermore, certain miR-NAs (epi-miR-NAs) counteract CpG methylation and regulate the components of epigenetic machinery, thus creating a tightly controlled feedback mechanism [5]. MiRNA signatures have been identified in NSCLC, not only demonstrating their biological role but also suggesting them as potential non-invasive diagnostic and therapeutic biomarkers in this type of lung malignancy [6].

Merkel cell polyoma virus (MCPyV) is a double stranded DNA virus that belongs to the family of human polyoma viruses. Several members of the human polyoma viruses have demonstrated oncogenic properties in cell culture and animal models however to date MCPyV is the only member associated with cancer in humans [7]. The prevalence of MCPyV in Merkel cell carcinomas (MCC) of the skin reaches 80%[7] while we and others have previously demonstrated the presence of MCPyV in patients with non-small cell lung cancer (NSCLC) [8]. We have shown that the deregulation of *Braf* and *Bcl*-*2* is associated with the presence of MCPyV DNA in NSCLC patients, implicating apoptotic pathways with polyoma virus infection in human lung cancer [9]. Although additional studies are needed for the accurate determination of the prevalence of MCPyV in NSCLC, it appears that the presence of this virus may be implicated with carcinogenic phenomena not only in the skin but also in malignancies of the lung [10].

Viruses are known to alter the expression of several host miRNAs in order to highjack cellular machinery for their propagation or to evade antiviral responses while several miRNAs associated with cancer, are also affected by viruses that cause tumours in humans [11]. Information regarding the effect of MCPyV infection on host miRNA expression is extremely limited [12]. Interestingly, MCPyV appears to suppress the expression of miR-203 though yet unknown mechanisms in MCPyV-positive MCC and this was directly linked to increased cell proliferation and evasion of cell cycle arrest [12].

Considering the emerging interactions between microRNA regulation and MCPyV infection in MCC, we hypothesised that the expression of NSCLC-associated microRNAs may be influenced by the presence of MCPyV in cancerous lung tissues. Thus, in this study, we investigated the expression profiles of the microRNAs previously associated with NSCLC such as miR-21, miR-367, miR-155, miR-146a, miR-302c, miR-145 and miR-376c and their corresponding target genes and associated pathways in samples from primary NSCLC that were positive or negative for MCPyV DNA. Our results demonstrated aberrant expression patterns of a miR-21, miR-376c and miR-145 which correlated with the deregulated expression of several target genes, providing novel evidence of a MCPyV induced epigenetic mechanism in NSCLC.

## RESULTS

### Polyomavirus detection

Initially we tested the NSCLC tissue samples for the presence of MCPyV DNA. Eight (N=8) NSCLC samples and five of the non-malignant corresponding controls (N=5) tested positive for MCPyV DNA. Sixteen (N=16) NSCLC samples and five of the non-malignant corresponding controls (N=5) tested negative for MCPyV genomes. From our original cohort [8] we were able to obtain non-malignant appropriate control tissues from 5 NSCLC patients positive for MCPyV and from 5 NSCLC patients negative for MCPyV. Demographics, biopsy results and malignant subtypes of NSCLC of patients are shown in Table 1. The two groups of MCPyV-positive and –negative NSCLC samples were matched for age, gender, smoking history and NSCLC subtypes since no significant differences were observed between the two groups.

**Table 1:** The clinicopathological characteristics and histological NSCLC types of all patients

Characteristics	MCPyV-positive	MCPyV-negative	P-value
Number	8	16	
Age^*^	68.00±8.21	62.82±11.63	NS
Sex (male/female)^**^	7/1	14/2	NS
Non-smokers^**^	1	1	NS
Smokers^**^	6	8	NS
Ex-smokers^**^	1	7	NS
Adenocarcinoma	5	10	
Squamous	2	5	
Large Cell Undifferentiated	1	1	

Values are expressed as means ± SD (standard deviation).

^*^t-test; *P* < 0.05 is considered statistically significant.

^**^χ^2^test; *P* < 0.05 is considered statistically significant.

NS; not significant.

The histological types consisted mainly of adenocarcinomas and squamous cell carcinomas. The two predominant histological MCPyV-positive types showed similarities in mean age of the patients.

### microRNA expression

Next, we compared the expression of the microRNAs miR-21, miR-145, miR-146a, miR-155, miR-302c, miR-367 and miR-376c in the MCPyV+ve and MCPyV-ve NSCLC samples and their corresponding control samples. MiR-367, member of a cluster that reprograms fibroblasts to induced pluripotent stem cells [13], and miR-145, a hypothesized tumor suppressor [14], have been shown to be associated with an unfavourable prognosis in resected NSCLC [15], while miR-145 and miR-146a, a mediator of inflammation [16], have been proposed as serum diagnostic biomarkers in NSCLC [17]. MiR-155, a multifunctional microRNA involved in haematopoietic lineage differentiation, immunity and viral infections, inflammation, cancer, and cardiovascular diseases [18], has been hypothesized to induce the development of NSCLC in patients from Asia and America [19]. Mir-376c suppresses non-small-cell lung cancer cell growth and invasion by targeting LRH-1-mediated Wnt signaling pathway [20]. MiR-302c has been proposed to prevent Ras-induced senescence by inhibiting p21 [21], while miR-21, the most upregulated microRNA in solid tumours, has been shown to be associated with NSCLC prognosis [22], predict recurrence and unfavourable survival in NSCLC [23] and sensitivity to ionizing radiation [24]. Normalisation was performed using miR-191 as it was found to be the most stably expressed microRNA in FFPE lung samples, as previously suggested [15].

**Figure 1:**
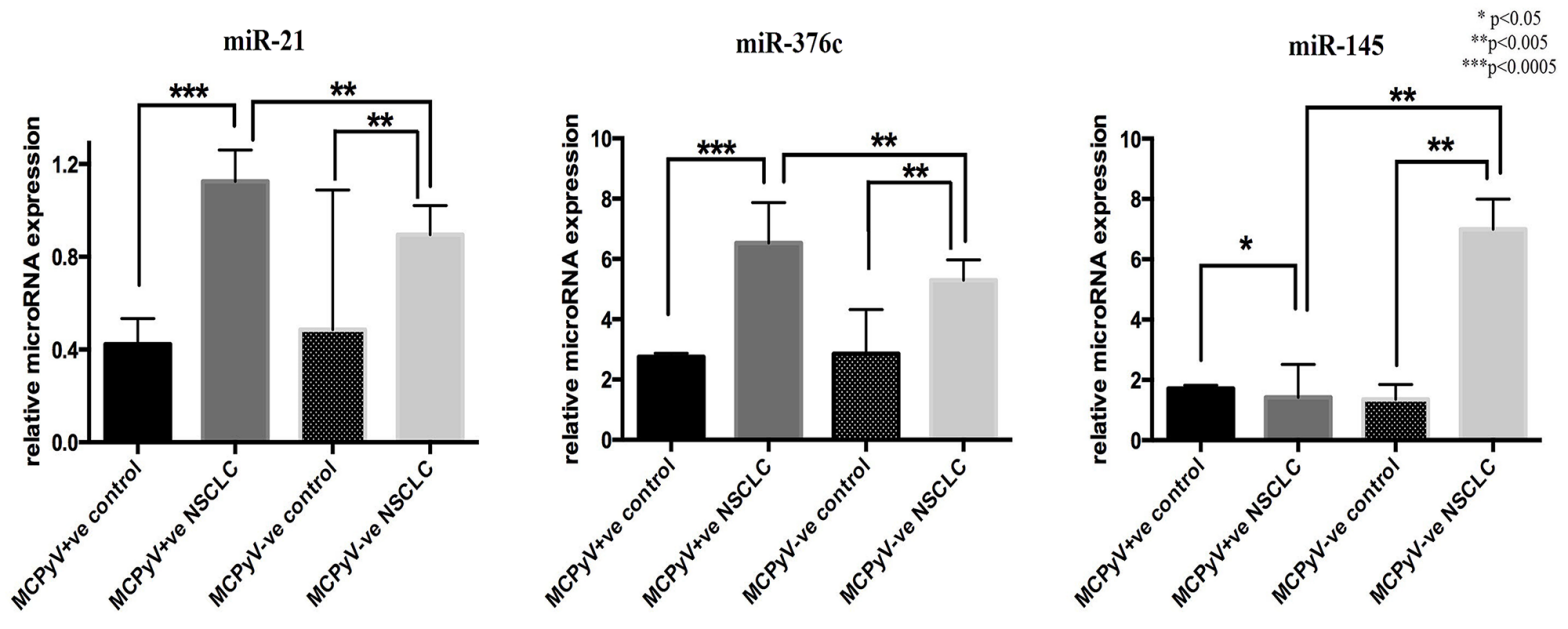
Expression profile of microRNAs in lung tissue samples of NSCLC patients (MCPyV+ve N=8, MCPyV-ve N=16) and controls (MCPyV+ve N=5, MCPyV-ve N=5) in relation to the presence or absence of MCPyV Samples showed normal distribution, One-way Anova and confirmatory student's t test were used for the analysis. ^*^ p<0.05, ^**^ p<0.005, ^***^ p<0.0005.

MiR-21 and miR-376c were significantly overexpressed in the NSCLC samples compared to the controls (Table 2, Figure 1). MiR-21 and miR-376c were also significantly higher in MCPyV-positive NSCLC samples as compared to MCPyV-negative samples (Table 2, Figure 1). Alternatively, miR-145 was significantly overexpressed in MCPyV-negative NSCLC samples compared to MCPyV-positive NSCLC samples and the control non-malignant tissue samples (Figure 1). The expression of miR-367, miR-155, miR-146a and miR-302c showed no difference either between the MCPyV-positive and the MCPyV-negative NSCLC samples or NSCLC and control samples.

**Table 2:** Expression profiles of microRNAs in lung tissue samples of NSCLC patients and controls in relation to the presence or absence of MCPyV

MEAN±SD	MCPyV +ve controls	MCPyV +ve samples	MCPyV -ve controls	MCPyV -ve samples	*P*-value
miR-21	0.43±0.24	1.13±0.08	0.49±0.60	0.88±0.05	0.013
miR-376c	2.75±0.26	6.53±0.38	2.85±1.47	5.29±2.51	0.048
miR-145	1.71±0.23	1.35±0.21	1.28±0.43	6.99±3.99	0.0002

Values are expressed as means ± SD (standard deviation).

*P* < 0.05 is considered statistically significant.

NS; not significant.

No significant associations were found between gene expression and age, gender, smoking history or NSCLC subtype. The survival curve of both groups did not differ significantly.

### Gene expression

We further sought to investigate the expression profiles of genes targeted by the dysregulated microRNAs in this cohort.

**Figure 2:**
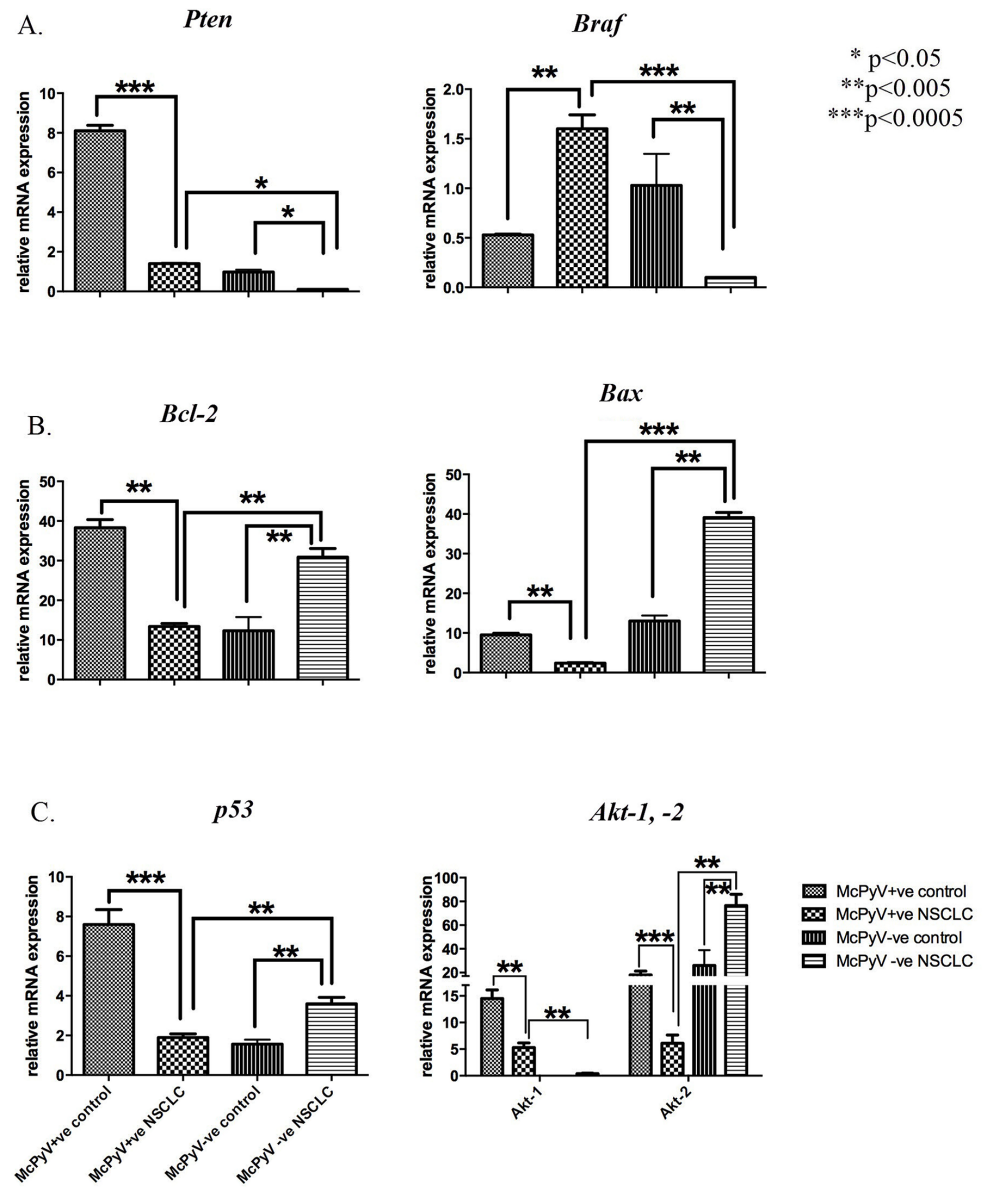
Expression profile of miR-21 target genes and associated pathways’ genes *Pten, Braf, Bcl-2, Bax, p53, Akt-1, Akt-2* in lung tissue samples of NSCLC patients (MCPyV+ve N=8, MCPyV-ve N=16) and controls (MCPyV+ve N=5, MCPyV-ve N=5) in relation to the presence or absence of MCPyV Samples showed normal distribution, One-way Anova and confirmatory student's t test were used for the analysis. ^*^ p<0.05, ^**^ p<0.005, ^***^ p<0.0005.

MiR-21 target genes were categorised, mainly based on the hallmarks of cancer [25], a wheel of potential genes targeted by miR-21 in a tumorous environment as well as the potential pathways influenced by miR-21. The *Pten* and *Braf* genes, involved in the sustenance of proliferative signalling, were significantly underexpressed and overexpressed, respectively in the MCPyV-positive NSCLC samples compared to their corresponding controls (Table 3, Figure 2A). *Bcl-2* and *Bax*, anti- and pro-apoptotic genes, respectively, implicated in resistance to cell death, were underexpressed in the MCPyV-positive NSCLC samples compared to the MCPyV-negative NSCLC samples as well as to the controls (Figure 2B). Genes implicated in the deregulation of genomic stability, *p53* and *Akt2*, were underexpressed in the MCPyV-positive NSCLC samples compared to controls and the MCPyV-negative NSCLC samples (Figure 2C), while *Akt1* was underexpressed in the MCPyV-positive NSCLC samples compared to the controls but overexpressed compared to the MCPyV-negative NSCLC samples.

**Table 3:** Expression profiles of microRNAs’ target genes in lung tissue samples of NSCLC patients and controls in relation to the presence or absence of MCPyV

Gene expression profiles				
MEAN±SD	MCPyV +ve controls	MCPyV +ve NSCLC samples	MCPyV –ve controls	MCPyV -ve NSCLC samples
**miR--21 targets**				
Direct target genes				
*Pten*	8.10±0.8	1.4±0.02	0.98±0.32	0.10±0.00
*Bcl-2*	38.30±5.8	13.40±2.10	12.32±9.87	40.90±6.10
*Daxx*	10.08±1.23	16.79±8.10	8.69±3.63	7.10±0.81
*Pkr*	357.1±56.04	64.71±11.37	41.97±6.54	46.71±23.71
*Timp3*	6.10±1.31	1.4±0.02	0.03±0.00	0.30±0.00
Associated pathways’ genes				
*Braf*	0.53±0.02	1.60±0.40	1.03±0.94	0.10±0.00
*Bax*	9.50±1.20	2.4±0.31	13.69±4.12	39.1±3.72
*p53*	7.60±2.10	1.90±0.51	1.56±0.65	3.6±0.89
*Akt-1*	14.50±1.63	5.30±0.85	0.03±0.00	0.4±0.09
*Akt-2*	18.10±3.12	3.10±1.56	26.45±13.58	76.5±9.54
*Hif1a*	11.71±0.98	78.80±6.57	6.91±2.23	3.90±0.09
**miR--376c targets**				
*Alk7*	1.13±0.76	0.22±0.32	0.58±1.21	0.62±0.06
*Mmp9*	357.1±56.04	64.71±11.37	123.89±55.63	166.71±15.71
*Grb2*	12.31±0.32	1.98±0.02	5.32±1.29	6.32±0.34
**miR--145 targets**				
*Oct4*	0.87±0.02	1.10±0.41	0.49±0.02	0.54±0.26
*Sox2*	0.92±0.38	5.21±2.78	2.65±0.03	1.21±0.72
*Fascin1*	-	53.16±12.28	-	28.04±6.89

Values are expressed as means ± SD (standard deviation).

NS: non significant.

**Figure 3:**
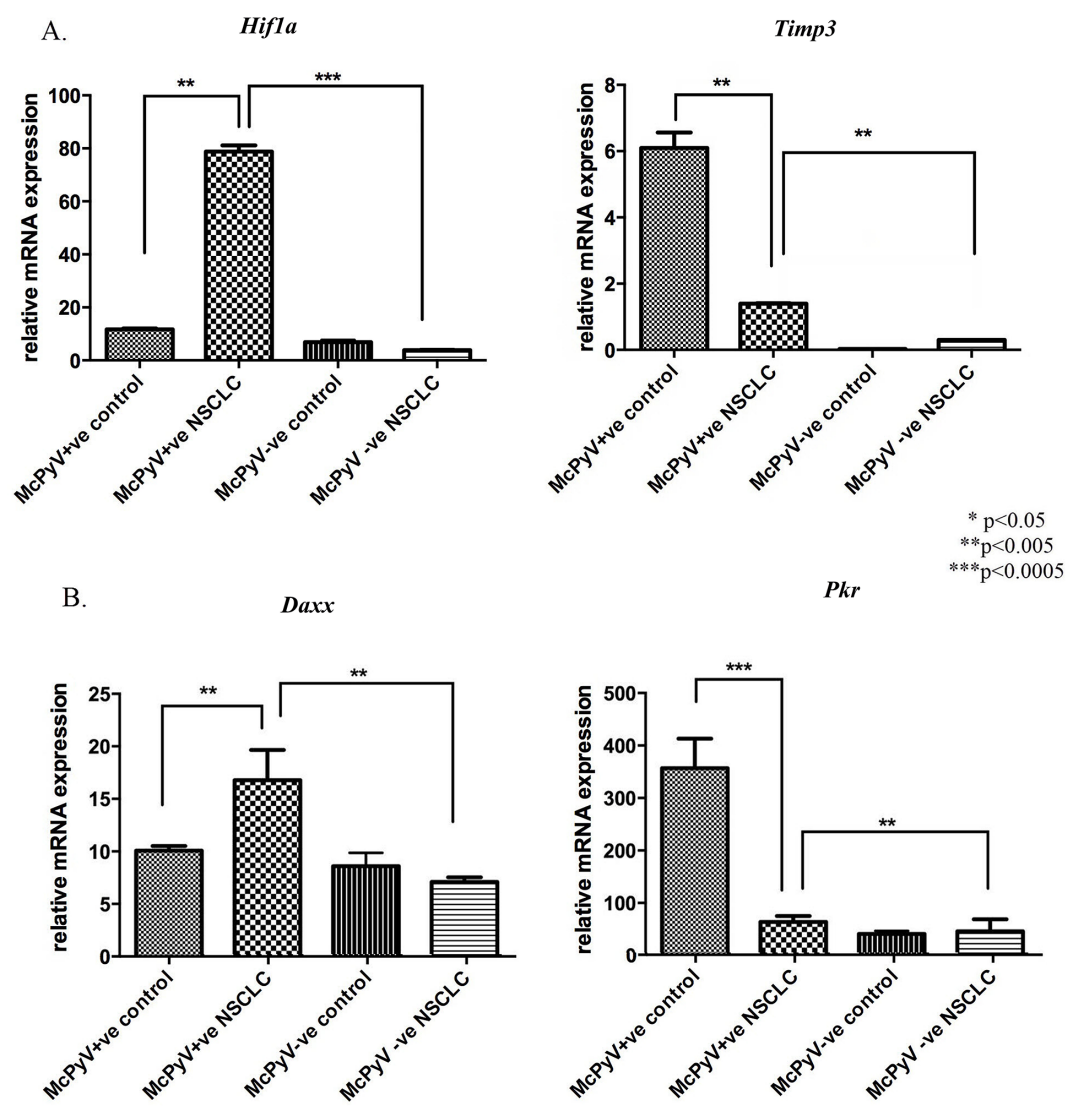
Expression profile of miR-21 target genes and associated pathways’ genes *Hif1a, Timp3, Daxx, Pkr* in lung tissue samples of NSCLC patients (MCPyV+ve N=8, MCPyV-ve N=16) and controls (MCPyV+ve N=5, MCPyV-ve N=5) in relation to the presence or absence of MCPyV Samples showed normal distribution, One-way Anova and confirmatory student's t test were used for the analysis. ^*^ p<0.05, ^**^ p<0.005, ^***^ p<0.0005.

Overexpression was detected for the hypoxia-associated and innate immune response-inducible transcription factor *Hif1a* in the MCPyV-positive NSCLC samples compared to the controls and the MCPyV-negative NSCLC samples (Figure 3A), while the tumor-promoting inflammatory gene *Daxx* was overexpressed in the MCPyV-positive NSCLC samples compared to the controls as well as the MCPyV-negative NSCLC samples (Figure 3B). Angiogenic deregulator *Timp3* was underexpressed in the MCPyV-positive NSCLC samples compared to the controls but overexpressed compared to the MCPyV-negative NSCLC samples (Table 3, Figure 3A). Lastly, *Pkr*, an antiviral innate immune response-inducible gene also involved in tumor-promoting inflammatory pathways, was expressed at significantly higher levels in the MCPyV-positive tissue samples compared to the MCPyV-negative tissues (Table 3, Figure 3B). Interestingly, the expression of *Pkr* was significantly suppressed in the malignant tissues.

All miR-21 direct target genes showed a significant negative correlation with miR-21 expression in the MCPyV-positive NSCLC group, apart from *Daxx* which showed a positive correlation (Table 4). *Pten, Bcl-2* and *Pkr* showed moderate negative correlation with miR-21 expression in MCPyV-positive NSCLC, while *Daxx* and *Timp3* showed a weaker correlation with miR-21 expression in MCPyV positive NSCLC.

**Table 4:** Correlations between microRNAs and their target genes in lung tissue samples of NSCLC patients and controls in relation to the presence or absence of MCPyV

Correlations				
r/r^2^/p value	MCPyV +ve controls	MCPyV +ve NSCLC samples	MCPyV -ve controls	MCPyV -ve NSCLC samples
**Correlation of target genes with miR--21**				
*Pten*	NS	r = -0.6205, r^2^ =0.3850, p = 0.0009	NS	r = -0.4027, r^2^ = 0.1621, p = 0.0460
*Bcl-2*	r = +0.5603, r^2^ =0.3139, p =0.0036	r = -0.5044, r^2^ =0.2544, p = 0.0101	NS	NS
*Daxx*	NS	r = +0.4309, r^2^ =0.1857, p = 0.0315	NS	r = -0.2398, r^2 ^= 0.2598, p = 0.0047
*Timp3*	NS	r = -0.4293, r^2^ =0.1843, p = 0.0322	r = -0.8293, r^2^ =0.5845, p =0.0432	r = -0.1493, r^2^ = 0.3649, p = 0.0212
*Pkr*	NS	r = -0.4946, r^2^ =0.2446, p = 0.0266	NS	r = -0.9746, r^2^ = 0.6597, p = 0.0023
**Correlation of target genes with miR--376c**				
*Alk7*	NS	r = -0.6951, r^2^ =0.5912, p = 0.0251	NS	NS
*Mmp9*	r = +0.4351, r^2^ =0.1964, p=0.0387	r = -0.3416, r^2^ =0.4108, p = 0.0023	NS	r = +0.6941, r^2 ^= 0.4679, p = 0.0039
*Grb2*	r = +0.6398, r^2 ^=0.2415, p =0.0289	r = -0.7589, r^2^ =0.6341, p = 0.0049	NS	NS
**Correlation of target genes with miR--145**				
*Oct4*	NS	r = +0.2341, r^2^ = 0.3019, p = 0.0056	r =+0.1956, r^2^ =0.2098, p =0.0042	r = -0.6832, r^2 ^= 0.5912, p = 0.0183
*Sox2*	NS	r = +0.3965, r^2 ^= 0.4170, p = 0.0038	NS	r = -0.9230, r^2^ = 0.7154, p = 0.0058
*Fascin1*	-	r = +0.6231, r^2 ^= 0.5439, p = 0.0008	-	r = -0.2206, r^2^ = 0.1905, p = 0.0029

NS: non significant, r=Pearson's r coefficient, r^2^=coefficient of determination.

**Figure 4:**
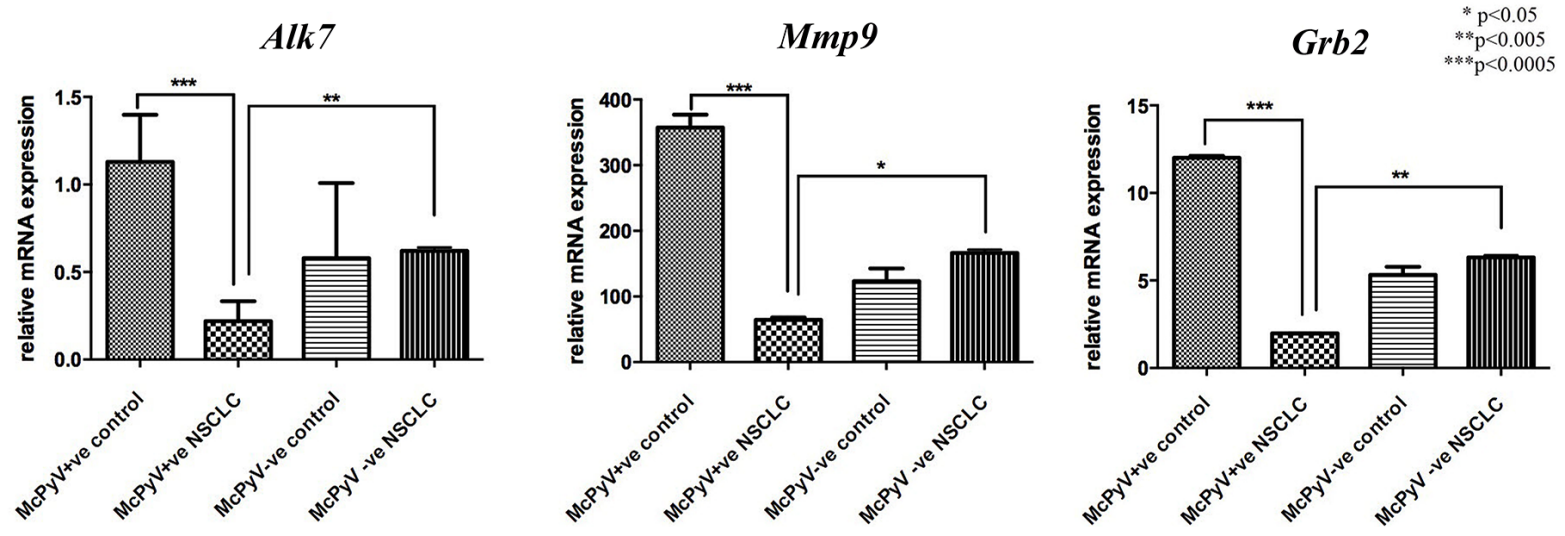
Expression profile of miR-376c target genes *Alk7, Mmp9, Grb2* in lung tissue samples of NSCLC patients (MCPyV+ve N=8, MCPyV-ve N=16) and controls (MCPyV+ve N=5, MCPyV-ve N=5) in relation to the presence or absence of MCPyV Samples showed normal distribution, One-way Anova and confirmatory student's t test were used for the analysis. ^*^ p<0.05, ^**^ p<0.005, ^***^ p<0.0005.

The transcript levels of genes targeted by miR-376c, *Alk-7, Mmp9* and G*rb2* were significantly decreased in the MCPyV-positive NSCLC samples compared to the controls and MCPyV-negative samples (Table 3, Figure 4). *Alk7* and *Grb2* strongly negatively correlated with miR-367c in the MCPyV-positive NSCLC group (Table 4), while *Mmp9* showed a weak negative correlation with miR-376c expression in MCPyV-positive NSCLC.

**Figure 5:**
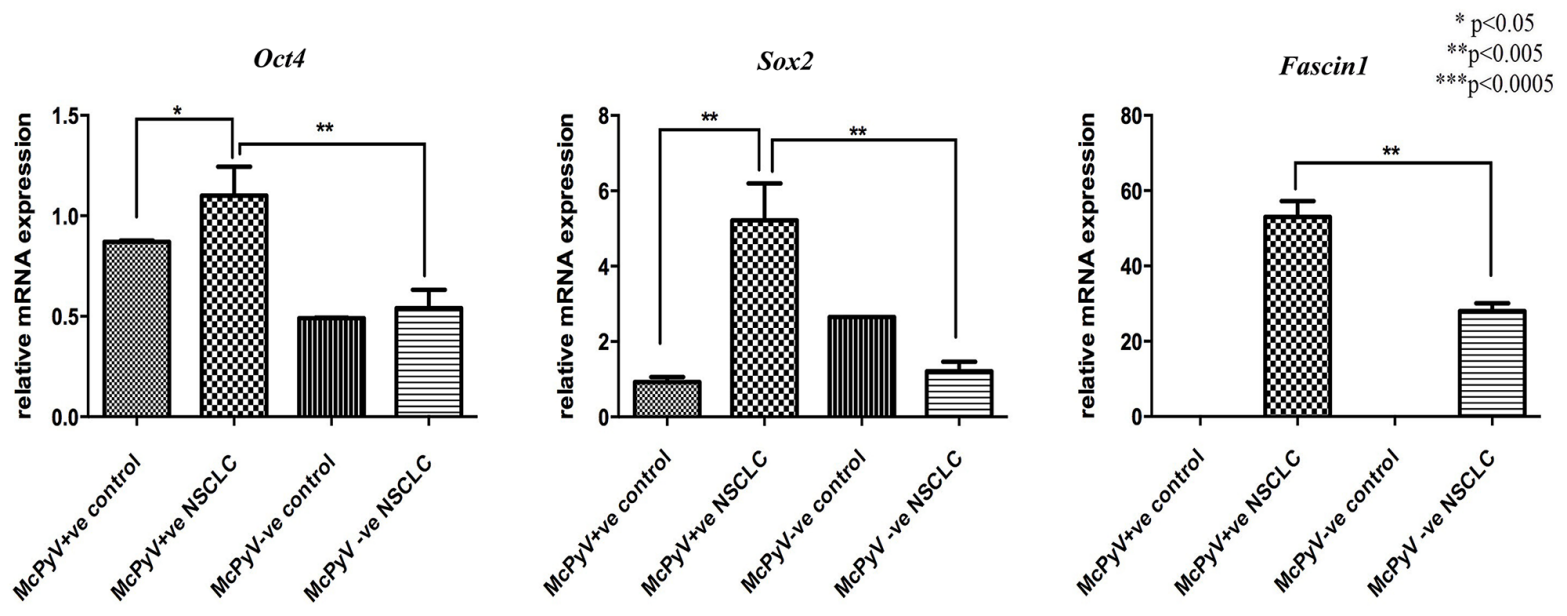
Expression profile of miR-145 target genes *Oct4, Sox2, Fascin1* in lung tissue samples of NSCLC patients (MCPyV+ve N=8, MCPyV-ve N=16) and controls (MCPyV+ve N=5, MCPyV-ve N=5) in relation to the presence or absence of MCPyV Samples showed normal distribution, One-way Anova and confirmatory student's t test were used for the analysis. ^*^ p<0.05, ^**^ p<0.005, ^***^ p<0.0005.

MiR-145 targets genes *Oct4, Sox2* and *Fascin1* were overexpressed in the MCPyV-positive NSCLC samples compared to the controls and the MCPyV-negative samples (Table 3, Figure 5). *Oct4, Sox2* and *Fascin1* negatively correlated with miR-145 in the MCPyV-positive NSCLC group (Table 4), *Oct4* and *Sox2* had a weak correlation result, while *Fascin1* showed a moderate to strong correlation coefficient.

The *Akt-1* transcript levels correlated significantly with the smoking status only in the MCPyV-negative group. No other significant correlation between microRNA target gene expression and clinical parameters arose.

## DISCUSSION

The present study aimed to investigate the potential association between human polyomavirus infection and epigenetic alterations involving the expression of microRNAs in NSCLC. Among the evaluated microRNAs, miR-21 and miR-376c showed a modest but significant increased expression in MCPyV-positive NSCLC samples relative to the MCPyV-negative tumour samples. Conversely, miR-145 was strongly downregulated in the MCPyV-positive NSCLC samples relative to the MCPyV-negative tumour samples.

### Tumour aggressiveness

Several miR-21 target genes and associated pathways are implicated in the majority of the hallmarks of cancer [25]. In both MCPyV-positive and MCPyV-negative NSCLC samples, we found high miR-21 levels that were correlated with low *PTEN* mRNA levels. This inverse association was stronger in the MCPyV-positive tumour samples and may indicate a poor Tyrosine Kinase Inhibitor (TKI) resistance clinical response and a shorter survival [26]. This should be taken into consideration, in combination with data suggesting that the downregulation of miRNA-21 could sensitize radioresistant NSCLC cells by inhibiting cell proliferation and enhancing apoptosis through the inhibition of the PI3K/Akt signalling pathway [24]. *Braf* gene involved in sustaining pro-proliferative signalling was found strongly upregulated in the MCPyV-positive NSCLC samples relative to the MCPyV-negative tumour samples as previously shown [8]. The combination of low *Pten* levels with high *Braf* expression provides evidence for increased tumour aggressiveness associated with MCPyV in NSCLC.

We also investigated the *Bax/Bcl-2* equilibrium, which is related to apoptosis resistance in association with miR-21 expression, following up on our previous study [8]. *Bax*, widely described as a pro-apoptotic factor, and *Bcl-2*, representative of anti-apoptotic proteins, were found underexpressed in MCPyV-positive NSCLC cases, both negatively correlating with miR-21. A ratio of *Bax/Bcl-2* lower than 1, as was observed in the MCPyV-positive samples, indicates hyperexpression of *Bcl-2* and loss of *Bax* and has been shown to positively correlate with the severity of the preneoplastic lesions, from low-grade to high-grade [27].

Importantly MCPyV-positive NSCLC samples showed decreased *p53* expression relative to the MCPyV-negative tumour samples. Inhibition of *p53* activity is a characteristic of polyoma viruses including MCPyV [28]. Recent data suggest that the inhibition of miR-21 would be beneficial in apoptosis-inducing cancer therapies directed against p53-deficient tumours [29].

It has been suggested that loss of angiogenic deregulator, promotes tumor malignancy and subsequent relapse and poor survival in patients with HPV-infected non-small cell lung cancer [30]. In the present study, MCPyV-infected NSCLC cells showed a significant underexpression of *Timp3* compared to the controls. Taken together, these results may hint at a poorer outcome in the presence of MCPyV DNA in NSCLC patients.

MiR-376c, was significantly overexpressed in MCPyV-positive NSCLC samples, and negatively correlated with its target genes, *Alk7, Mmp9* and *Grb2*, in MCPyV-positive NSCLC samples. MiR-376c has been suggested to inhibit *Alk7* expression, cell proliferation and apoptosis only in ovarian cancer cell lines [31], and lead to poor chemotherapy response. *Mmp9*, known to promote lung tumour metastasis [32], has been suggested to be inhibited by miR-129 [33] and has been proposed as a possible therapeutic target [33]. In addition, cytosolic adaptor protein GRB2, a critical mediator of oncogenic EGFR signaling through activation of RAS [34], is required for survival of cells with mutant EGFR [35]. To the best of our knowledge, this is the first report of *Mmp9* miR-376c mediated inhibition in human NSCLC, as well as miR-376c mediated *Grb2* downregulation in MCPyV-positive tumour samples.

Importantly, miR-145, a suggested tumor-suppressor [36] was strongly downregulated in MCPyV-positive NSCLC compared to MCPyV-negative NSCLC, while it was significantly higher in MCPyV-negative NSCLC relative to the non-malignant controls. This striking difference was also reflected in the miR-145 target genes, *Oct4/Sox2/Fascin1* which were all upregulated in the MCPyV-positive tumour samples. It has been shown that miR-145 expression is negatively correlated with the levels of Oct4/Sox2/Fascin1 in lung adenocarcinoma and an Oct4(high)/Sox2(high)/Fascin1(high)/miR145(low) phenotype, as in the present study, predicted poor prognosis [37]. Moreover, it was also revealed that the repressive effect of miR-145 on tumour metastasis was mediated by inhibiting the epithelial-mesenchymal transdifferentiation (EMT) and metastastic ability, partially by regulating Oct4/Sox2/Fascin1 [37].

### Innate anti-virus response

In the present study, miR-21 target genes and associated pathways involved in anti-viral response, type I interferon-inducible genes *Daxx*, *Pkr*, as well as *Hif1a*, were analysed in NSCLC cases. *Daxx* and *Hif1a* expression increased in MCPyV positive NSCLC while it correlated positively with miR-21 expression, suggesting that *Daxx* expression was not directly regulated by miR-21 in MCPyV positive NSCLC. *Pkr* was upregulated in MCPyV positive non-malignant tissues and not in MCPyV positive NSCLC tissues where it negatively correlated with miR-21 expression. Implication of *Daxx* and *Hif1a* in DNA hypermethylation and apoptosis [38] has recently emerged, while *Hif1a* overexpression, as in MCPyV-positive NSCLC samples, has been associated [39] with the formation of cancer-stem-like-like cells undergoing a process of endothelial-mesenchymal transition, both of which are inhibited by miR-21. In the present study, *Daxx* and *Hif1a* expression correlated positively with miR-21 expression in NSCLC, suggesting a miR-21-independent expression in NSCLC. *Pkr*, which negatively correlated with miR-21 overexpression in MCPyV-positive NSCLC specimens, has been suggested to be markedly involved in the development of NSCLC and may serve as a potential prognostic marker for patients with this deadly disease [40]. *Pkr* was upregulated only in non-malignant tissues and not in NSCLC tissues. This may suggest a possible *Pkr* inhibition in tumour cells which would inhibit the arrest in cellular translation processes and subsequently result in both virus and tumour cell resistance to a potent intrinsic cellular defence mechanism.

In conclusion, the present study explored the epigenetic alterations in lung tumor cells caused by the presence of MCPyV DNA in NSCLC patients. The expression profiles of microRNAs and their target genes in this study leads to the hypothesis that the presence of Merkel Cell Polyoma virus may lead to a more aggressive NSCLC as well as poorer outcomes in NSCLC prognosis. As a single miRNA may inhibit up to several hundred mRNAs, aberrant miRNA expression may suppress a multitude of transcripts and profoundly influence cancer-related signalling pathways; thus, these results must be interpreted with caution. Further research, particularly on therapeutic interventions is essential in delineating epigenetic mechanisms implicated in this deadly disease.

## MATERIALS AND METHODS

### Patients

The lung tissue patient group comprised of 24 consecutive patients with non-small cell lung cancer (NSCLC) from the Department of Thoracic Medicine, University Hospital of Heraklion, Crete, Greece. The patients included in this study were classified according to the criteria of WHO (1997). The tissue control group consisted of 10 samples from macroscopically healthy sites of the lung derived from NSCLC patients included in this cohort and were histologically verified. Demographics and lung carcinoma subtypes of all patients are summarized in Table 1.

### Ethics statement

The Ethics Committee of the University Hospital of Heraklion, Crete approved the protocol (Reg. No 140/4-2-2015) and all patients and control subjects provided informed consent in written form.

### Biological samples and processing

Twenty-four lung cancer tissue specimens and 10 non-malignant specimens from corresponding patients were obtained from paraffin-embedded blocks from the Laboratory of Pathology, University Hospital of Heraklion, Crete. All the samples were obtained from surgical resections.

### MCPyV detection, MicroRNA and mRNA expression

Twenty four paraffin-embedded tissue specimens from NSCLC patients and ten corresponding controls (from macroscopically and histologically verified healthy sites of respective appropriate controls from 5 NSCLC patients positive for MCPyV and from 5 NSCLC patients negative for MCPyV in this cohort) were processed using RecoverAll™ Total nucleic acid isolation from Ambion, for the isolation of total RNA as recommended by the manufacturer. RNA concentration and purity were evaluated by a spectrophotometer (Nanodrop).

The presence of MCPyV DNA was tested using Real-Time quantitative PCR protocols, using the MCV138 set of primers, which targets the Large T antigen (LTA) region, as previously described [8]. The integrity and quality of the extracted DNA were confirmed after the successful amplification of the *beta2-microglobulin* gene in all samples.

Particular care was taken and all manipulations were performed inside a PCR-hood to avoid potential contamination. Each PCR reaction contained two negative controls. DNA from a Merkel cell carcinoma patient served as the positive control. All Real-Time PCR reactions were carried out in an Mx3000P Real Time PCR system while additionally, PCR end-products were analyzed on a 2% agarose gel, stained with ethidium bromide and the bands were visualized under a UV transilluminator (260 nm). These viral PCR products were then subjected to direct sequencing analysis to verify the specific amplification of the MCPyV LTA region.

For the analysis of microRNA expression levels, 10ng total RNA were used in reverse transcriptase and Real-Time qPCR reactions using the TaqMan™ microRNA assays (Life Technologies) and 7500 Fast Real-Time PCR system (Applied Biosystems). Reverse transcription was performed at 16°C for 15 min, 42°C for 30 min, 85°C for 5 min and 2fold diluted cDNA samples were analysed by Real-Time PCR (95°C for 10 min, followed by 40 cycles of 95°C for 15 sec, 60°C for 1 min). Probe and primer sequences are summarized in Table 5. miR-191 was used as endogenous control for the normalization of microRNA expression levels in all samples, due to its high stability and consistency, as previously described [14, 41].

**Table 5:** Primer sequences used for quantitative Real-Time RT-PCR

Gene	Primer pair sequence (5’-3’)	Annealing Temperature
*GAPDH*	FOR: AGCCACATCGCTCAGACAC REV : GCCCAATACGACCAAATCC	54 °C
*Braf*	FOR: AGAAAGCACTGATGATGAGAGG REV: GGAAATATCAGTGTCCCAACCA	58 °C
*Bcl-2*	FOR: GAAACCCCTAGTGCCATCAA REV: GGGACGTCAGGTCACTGAAT	55 °C
*p53*	FOR: GTGAGCGCTTCGAGATGTTC REV: ATGGCGGGAGGTAGACTGAC	60 °C
*Akt-1*	FOR: GCAGCACGTGTACGAGAAGA REV: GGTGTCAGTCTCCGACGTG	54 °C
*Akt-2*	FOR: CTCACACAGTCACCGAGAGC REV: TGGGTCTGGAAGGCATACTT	56 °C
*Bax*	FOR: TTCTGACGGCAACTTCAACTG REV: TTGGTGCACAGGGCCTGTAATC	61 °C
*Daxx*	FOR: CATGCGAGGTTCTGAGAATTG REV:GAGGAAGTGGTGGGGATTTC	54 °C
*Pten*	FOR:CGGCAGCATCAAATGTTTCAG REV:CGGCAGCATCAAATGTTTCAG	55 °C
*Pkr*	FOR: CCATGGGGAATTACATAGGC REV: CTTTCTGTCCCATTTTGCATT	52 °C
*Hif1a*	FOR: CTTGCATGGCTCTCAGATTCAC REV: AGAGGACAAGCAGATTCAAGGTG	60 °C
*Timp3*	FOR: GGCGGCAGCAGCGGCAATGAC REV: TACCAGCTTCTTCCCCACCACCTT	60 °C
*Grb2*	FOR: CCATCGCCAAATATGACTTCAAA REV: CTTCGTTCAAAACCTTGAGGATGT	62 °C
*Alk7*	FOR: ATGACCCGGGCGCTCTGCTCA REV: ATACTGTCAGCATCGCAGCTA	58 °C
*Mmp9*	FOR: ACTTTGACAGCGACAAGAGGTG REV: CCGGCACTGAGGAATGATCTAA	64 °C
*Oct4*	FOR: AGGGAAGGAGATTATGGAGGAGG REV: AGCTTTGGGCTCTGGAAGGC	60 °C
*Sox2*	FOR: AGGATAAGTACACGCTGCCC REV: TAACTGTCCATGCGCTGGTT	57 °C
*Fascin1*	FOR: CGCTCCAGCTATGACGTCTTCC REV: CAGGAGAACCTGCCTTTGATGTT	60 °C
**microRNA**	**microRNA Assay ID**	
Has-miR--191	002299	60 °C
Has-miR--21	000397	60 °C
Has-miR--145	000467	60 °C
Has-miR--146a	000468	60 °C
Has-miR--155	002623	60 °C
hsa-miR--302c-3p	000533	60 °C
Has-miR--367	000555	60 °C
Has-miR--376c	002122	60 °C

For gene expression analyses, 500ng of total RNA were first treated with DNAfree (Ambion) in order to remove genomic DNA contamination, followed by 1^st^ strand cDNA synthesis using Maxima RT ™ (Fermentas) and Real-Time qPCR analysis using Maxima SYBR-Green qPCRmix (Fermentas) on a Mx3005P qPCR system (Agilent Technologies). RT reaction was performed at 25°C for 10 min, 50°C for 20 min, 65°C for 10 min and 85°C for 5 min. 6fold diluted cDNA samples were analysed by Real-Time PCR (95°C for 10 min, followed by 40 cycles of 95°C for 15 sec, primer pair specific annealing temperature (Table 5)°C for 30sec, 72°C for 30sec, followed by 72°C for 10min). Transcript levels of validated miR-21 target genes (*Pten, Bcl-2, Daxx*, *Pkr*, *Timp3*, validated miR-376c target genes (*Grb2*, *Alk7*, *Mmp9*) and validated miR-145 target genes (*Oct4*, *Sox2*, *Fascin1*) obtained from TarBase.v7 were evaluated. We also evaluated the following genes associated with target genes and apoptotic pathways of miR-21 *p53* [42], *Braf* [43], *Akt-1, Akt-2* [44], *BaxX* [45], *Hif1a* [46]. *GAPDH* levels were used as endogenous control for the normalization of mRNA expression levels in all samples. Primers sequences and annealing temperatures are shown in Table 5.

### Statistical analysis

The Kolmogorov-SmiRnov test was used to determine whether the expression data obtained followed a normal distribution pattern. The mRNA expression of all the genes was compared between the groups of normal and pathological samples, as well as between groups with different histological features, using parametric procedures (Student's t-test and One way Anova), as all sample groups followed Gaussian (normal) distribution. Linear regression between microRNAs and their target genes were analysed with the Linear (Pearson) correlation test. Probability values (P-values) <0.05 were considered statistically significant. Statistical calculations were performed using SPSS 11.5 software (SPSS, Chicago, IL, USA).
